# Post-Weaning Diet Affects Faecal Microbial Composition but Not Selected Adipose Gene Expression in the Cat (*Felis catus*)

**DOI:** 10.1371/journal.pone.0080992

**Published:** 2013-11-28

**Authors:** Emma N. Bermingham, Sandra Kittelmann, Wayne Young, Katherine R. Kerr, Kelly S. Swanson, Nicole C. Roy, David G. Thomas

**Affiliations:** 1 Food Nutrition & Health, AgResearch Grasslands, Palmerston North, New Zealand; 2 Animal Nutrition & Health, AgResearch Grasslands, Palmerston North, New Zealand; 3 Division of Nutritional Sciences, University of Illinois at Urbana-Champaign, Urbana, Illinois, United States of America; 4 Department of Animal Sciences, University of Illinois at Urbana-Champaign, Urbana, Illinois, United States of America; 5 The Riddet Institute, Massey University, Palmerston North, New Zealand; 6 Centre of Feline Nutrition, Institute of Food, Nutrition and Human Health, Massey University, Palmerston North, New Zealand; Naval Research Laboratory, United States of America

## Abstract

The effects of pre- (i.e., gestation and during lactation) and post-weaning diet on the composition of faecal bacterial communities and adipose expression of key genes in the glucose and insulin pathways were investigated in the cat. Queens were maintained on a moderate protein:fat:carbohydrate kibbled (“Diet A”; 35:20:28% DM; n  =  4) or high protein:fat:carbohydrate canned (“Diet B”; 45:37:2% DM; n = 3) diet throughout pregnancy and lactation. Offspring were weaned onto these diets in a nested design (n  =  5 per treatment). Faecal samples were collected at wk 8 and 17 of age. DNA was isolated from faeces and bacterial 16S rRNA gene amplicons were analysed by pyrosequencing. RNA was extracted from blood (wk 18) and adipose tissue and ovarian/testicular tissues (wk 24) and gene expression levels determined using RT-qPCR. Differences (P<0.05) in composition of faecal bacteria were observed between pregnant queens fed Diet A or B. However, pre-weaning diet had little effect on faecal bacterial composition in weaned kittens. In contrast, post-weaning diet altered bacterial population profiles in the kittens. Increased (P<0.05) abundance of Firmicutes (77% vs 52% of total reads) and Actinobacteria (0.8% vs 0.2% of total reads), and decreased (P<0.05) abundance of Fusobacteria (1.6% vs 18.4% of total reads) were observed for kittens fed the Diet A compared to those fed Diet B post-weaning. Feeding Diet B pre-weaning increased (P<0.05) the expression levels of INRS, LEPT, PAI-1 and tended to increase GLUT1, while the expression levels of IRS-1 in blood increased in kittens fed Diet A pre-weaning. Post-weaning diet had no effect on expression levels of target genes. Correlations between the expression levels of genes involved in glucose and insulin pathways and faecal Bacteriodetes and Firmicutes phyla were identified. The reasons for why post-weaning diet affects microbial populations and not gene expression levels are of interest.

## Introduction

Mammalian health is driven by a complex interaction between the host genome, its diet and the composition and function of the microbiota that inhabit its intestine. The plasticity of the host genome is well established; it responds to changes in diet in part via epigenetic regulation of gene expression [1], [2]. Pre-weaning diet (gestation and lactation) is known to affect the function of the genome and can have long term effects on the health of offspring [3], [4]. While information is available on the effects of pre- and post-weaning diet on changes in gene expression [5], [6], less information is available about these periods on the composition of intestinal microbiota.

The stability of intestinal microbiota is of interest, due to the relationship between the diet and the metabolic health of the host [7]. The intestinal microbiota of adult cats can change in response to short-term diet [8], [9], however it has been suggested that in the long term intestinal microbiota profiles appear to be stable [10]. Diet-induced-changes in microbiota have been linked to impaired intestinal function (e.g., increased intestinal permeability [11]) giving rise to disorders such as obesity [12], due in part to the ability of the intestinal microbiota to extract more energy from the diet and make it available to the host [13]. Therefore, many studies have focussed on the host at weaning, as the intestinal microbiota is still developing and is pliable [14].

More recently it has been recognised that the microbiota present at birth have long-term impacts on an individual's health and has a potential role in the risk of disease [15]. It is typically accepted that the intestinal tract of the newborn is sterile [16], however there is some evidence to suggest that there is translocation of microbiota from the mother to the offspring before birth [17], [18]. Further, recent opinions suggest that maternal diet during gestation may influence the composition of the microbiota in the intestinal tract of the offspring at birth [19]. Despite this, there is a lack of direct evidence to examine the effects of maternal diet on the composition of the offspring’s microbiota, with only a few papers investigating how maternal nutrition impacts the microbiome of the offspring [20], [21], [22].

We hypothesised that pre-weaning diet (gestation and lactation) would alter the composition of faecal microbiota and the expression of genes in the glucose and insulin metabolic pathways offspring. In order to investigate the hypothesis, we selected diets that contained contrasting levels of protein, fat and carbohydrate, that have shown to promote large differences in faecal bacteria profiles of adult cats [9].

## Materials and Methods

### Animal Study

**Ethics statement.** The protocol for this study was approved by the Massey University Animal Ethics Committee (MUAEC 10/108). All cats used are owned by Massey University and were housed at the Centre for Feline Nutrition (Massey University, Palmerston North, New Zealand) according to the Animal Welfare (Companion Cats) Code of Welfare (2007).

**Diets.** Commercially available dry (kibbled; Diet A) and wet (canned; Diet B) diets were utilised in this study. Both diets were formulated to meet all nutrient requirements for growth, gestation and lactation according to the Association of American Feed Control Officials (AAFCO). Diets were analysed for moisture content using a convection oven at 105°C (AOAC 930.15, 925.10) and the ash residue using a furnace at 550°C (AOAC 942.05). Crude protein and crude fat were determined using the Leco total combustion method (AOAC 968.06) and acid hydrolysis/Mojonnier extraction (AOAC 954.02), respectively. Gross energy (kJ/g) was determined using bomb calorimetry. Crude fibre was determined using the gravimetric method (AOAC 978.10) and Nitrogen Free Extractables (NFE) by difference (Table 1).

**Table 1:** Macronutrient profile of a commercially available Association of American Feed Control Officials (AAFCO) feed tested maintenance diets fed to domestic short hair kittens (*Felis catus*).

Component	Diet A^1^	Diet B^2^
Dry Matter (DM; % as is)	92.9	31.7
Crude Protein (% DM)	35.3	45.3
Crude Fat (% DM)	20.2	37.6
Ash (% DM)	7.5	7.2
Crude Fibre (% DM)	1.8	1.5
NFE^3^ (% DM)	28.2	2.0
Gross energy (kcal/100 g DM)	507.0	618.1
Metabolisable energy^4^ (kcal/100 g DM)	393.8	485.1

1 Ingredient list of Diet A (from pack): Corn, chicken and chicken meal, chicken digest, maize gluten, chicken tallow, tuna meal, poultry and poultry meal, iodinised salt, vegetable oil.

2 Ingredient list of Diet B (from pack): Meat by-products and meat derived from chicken, lamb, beef, and mutton; gelling agent; minerals; vegetable oil, emulsifier; colouring; vitamins, chelating agents.

3 Nitrogen free extractables calculated by difference (100 - crude protein - crude fat - crude fibre - ash).

4 Determined using modified Atwater factors of: crude protein (3·5 kcal ME/g DM), crude fat (8·5 kcal ME/g DM), NFE (3·5 kcal ME/g DM).

**Animals and housing.** Eight queens (bred from 3 unrelated toms; mean age 5.3 ± 0.4 yr) were randomly allocated to one of two diets (n  =  4 per diet). The queens received either a Diet A or Diet B *ad libitum* throughout the study (Table 1). Each queen was mated with a single unrelated male fed either Diet A or Diet B. Once pregnancy was confirmed (approximately 7–8 wk of pregnancy), the queens were moved to individual, multi-layered digestibility cages, containing nesting boxes on an upper shelf [23]. One week before birth, a faecal sample was collected within 15 min of defecation, snap frozen in liquid nitrogen and then stored at –85°C until analysis.

In total, twenty kittens were born and were weighed within 24 hours (h) of birth, and every other day for 2 weeks (wk), and then at weekly intervals until the end of the study.

**Figure 1:**
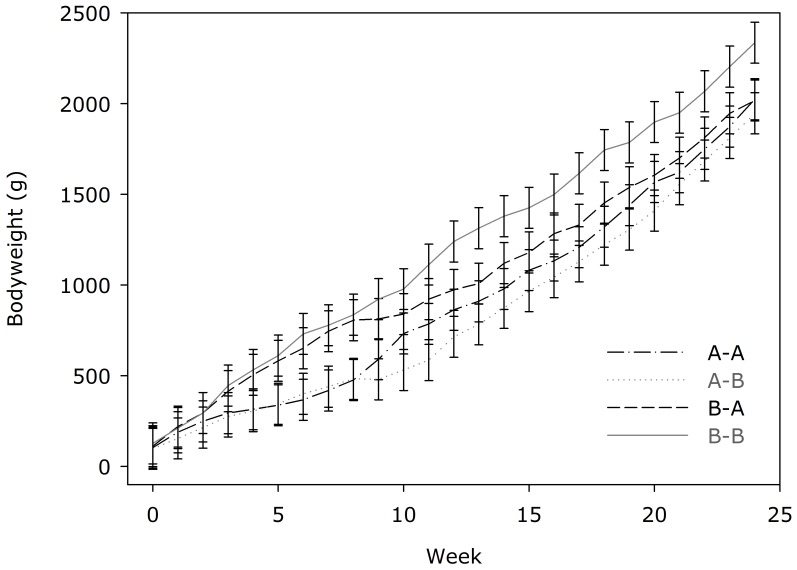
The effects of Diet A or Diet B on growth rates of kittens. Data are reported as means ± SEM.

From wk 0 to 4, all of the kittens received milk from their dam exclusively. Half of each litter was randomly assigned (within sex) onto Diet A and half onto Diet B, forming four dietary treatment groups (A-A, A-B, B-B, B-A; Table 2). At 4 wks of age, the kittens were weaned onto solid food in a gradual manner, receiving both the appropriate solid food and milk until wk 8, when the kittens were fully weaned. During the weaning process (4 wks onwards), kittens were group housed according to post-weaning diet for meals and socialisation, returning to their dam only for access to milk. By 8wks of age, kittens were housed exclusively according to their post-weaning diet. Diet and water were available *ad libitum* daily to allow for normal growth (Figure 1).

**Table 2:** Treatment groups for determining the effects of pre-weaning (*in utero* and during lactation) and post-weaning diet on intestinal microbiota in the domestic kitten (*Felis catus*; n = 5 per treatment).

Queen diet		Post-weaning diet	Post-weaning diet
		Diet A	Diet B
Pre-weaning diet	Diet A	A-A	B-A
		n = 3 females, n = 2 males	n = 3 females, n = 2 males
	Diet B	A-B	B-B
		n = 3 females, n = 2 males	n = 4 females, n = 1 male

Pre-weaning diet has been defined as the combined effects of gestation and lactation and early weaning, and the post-weaning phase as the point where the kitten is no longer receiving milk from its mother.

**Faecal sampling and preparation of barcoded amplification of bacterial 16S rRNA genes and amplicon pooling.** At 8 and 17 wks of age, the kittens were housed individually for 24 h and a fresh faecal sample was collected within 15 min of excretion, snap-frozen in liquid nitrogen and stored at –85°C.

Nucleic acids were extracted from faeces (30 mg) with a previously described combined bead-beating and phenol/chloroform protocol [24]. Fusion primers designed for GS FLX titanium pyrosequencing were used for microbiota analysis. Forward primers incorporating the Roche GS FLX adaptor A (5′-CCA TCT CAT CCC TGC GTG TCT CCG ACT CAG-3′), a unique 12-base molecular barcode [25], a two-base linker sequence and bacteria specific sequence Ba9F (5′-GAG TTT GAT CMT GGC TCA G-3′) [26], and a reverse primer incorporating adapter B (5′-CCT ATC CCC TGT GTG CCT TGG CAG TCT CAG-3′) and template specific sequence Ba515Rmod1 (5′-CCG CGG CKG CTG GCA C-3′) modified from Lane et al. [27] were used to amplify the bacterial 16S rRNA gene. Each PCR reaction contained 40 µl of Taq PCR MasterMix (Qiagen, Hilden, Germany), 28 µl reverse primer (0.6 µM) and 8 µl of barcoded forward primer (2 µM). A 19 µl aliquot of the reaction mix was transferred into a sterile tube to serve as no-template negative control. The remaining 57 µl were spiked with 3 µl of template DNA at a concentration between 20 and 40 ng µl^−1^. Amplification was performed using a Mastercycler proS (Eppendorf, Hamburg, Germany) with the following cycle parameters: initial denaturation at 95°C for 2 min, 30 cycles of denaturing (95°C, 20 s), annealing (52°C, 20 s) and elongation (72°C, 1 min), and a final 7-min extension at 72°C. Amplicons were purified using a High Pure PCR product purification kit (Roche Diagnostics, Mannheim, Germany) and sent to Macrogen (Seoul, Republic of Korea) for pyrosequencing using the GS FLX titanium system (Roche Diagnostics, Mannheim, Germany).

**Tissue gene expression.** At 18 wks of age a blood sample (0.5 ml) was taken and RNA extracted using the Total RNA Minikit (Blood/cultured cells) from Geneaid® (DNature, Gisborne, NZ) according to manufacturer’s instructions. At 24 wks of age, during neutering, samples of ovarian tissue (females only), testicular tissues (males only), and abdominal fat tissues (females only) were taken. The tissue samples were washed in ice-cold saline and then submersed in RNAlater® overnight, then stored at –20°C until analysis. RNA was extracted from abdominal fat and reproductive tissues using an RNeasy Plus Universal Mini Kit (QIAGEN Pty Ltd, VIC 3148, Australia) according to manufacturer’s instructions.

Expression of fatty acid synthase (FASN), Glucose transporter 1 (GLUT1) and 4 (GLUT4), insulin receptor (INSR), leptin (LEPT), hormone sensitive lipase (LIPE), uncoupling protein 2 (UCP2), insulin receptor substrate 1 (IRS-1) and 2 (IRS-2), plasminogen activator inhibitor-1 (PAI-1), peroxisome proliferative activated receptor-γ (PPARG) were determined in blood and tissue samples using RT-qPCR as described previously [6], [14], [28], [29]. Briefly, total RNA was reverse transcribed using Applied Biosystems High Capacity RNA-to-cDNA kits (Applied Biosystems Inc., Foster City, CA) and rt-qPCR on a Rotor-Gene 6000 thermocycler (Qiagen). The expression of FASN, GLUT1, GLUT4, INSR, IRS1, IRS2, LEPT, LIPE, PAL-1, UCP2 and PPARG were normalised against expression of GAPDH.

**Statistical Analyses.** Growth rates were determined utilizing repeated measures in the Mixed Model procedure of SAS using a two-way ANOVA with maternal and post-weaning diets as the main effects. The log transformation was utilized prior to analysis when necessary to maintain homogenous variance. No effect of gender was observed. Sequences were processed using the Quantitative Insights into Microbial Ecology (QIIME) version 1.5 pipeline [30]. Sequences were denoised and chimeric sequences removed using the ampliconnoise.py wrapper. Sequences passing quality checking were assigned to samples by their 12 bp barcodes, which were then binned into operational taxonomic units (OTU) at a minimum pair-wise similarity of 97%. Taxonomy was assigned to each OTU using the Ribosomal Database Project classifier using an 80% confidence threshold [31]. Beta diversity between samples at the minimum sequence depth attained (1640 sequences) was compared using weighted and unweighted UniFrac phylogenetic distances. Two factor (pre-weaning diet X post-weaning diet) multivariate analyses of community phylogenetic diversity was performed using the ADONIS function in the R package Vegan. Gene expression data was analysed two-way ANOVA with maternal and post-weaning diets as the main effects in R 2.14.1. Rank transformed bacterial taxa proportions from phyla and genera that made up > 0.05% of total bacteria in at least 5 samples were analysed by two-way ANOVA with maternal and post-weaning diets as the main effects. No effects of gender were observed. Results are reported as mean and associated pooled standard error of the mean (SEM) and were considered significant at P < 0.05 and a trend when 0.05 ≤ P ≤ 0.10. Correlation heat maps between microbiota abundance at the phylum level and gene expression generated using the CCA package (Ignacio González and Sébastien Déjean 2009. CCA: Canonical correlation analysis. R package version 1.2. http://CRAN.R-project.org/package=CCA).

## Results

### Influence of pre- and post-weaning diet on growth rates of kittens

Prior to weaning (wks 0–4), bodyweight was higher (P < 0.05) in kittens exposed to Diet B. Post-weaning, kittens fed Diet B were heavier (P < 0.05) than kittens fed Diet A (Figure 1).

Pyrosequencing of bacterial 16S rRNA gene barcoded amplicons resulted in a total of 120,520 sequences after denoising and chimera removal, with an average number of 2,564 sequences per sample (range  =  1,640 to 4,813). The number of OTUs identified was 1968. Sequence length was on an average of 481 bp (range  =  201 to 530 bp).

### Influence of diet on faecal bacterial community composition of queens

**Figure 2:**
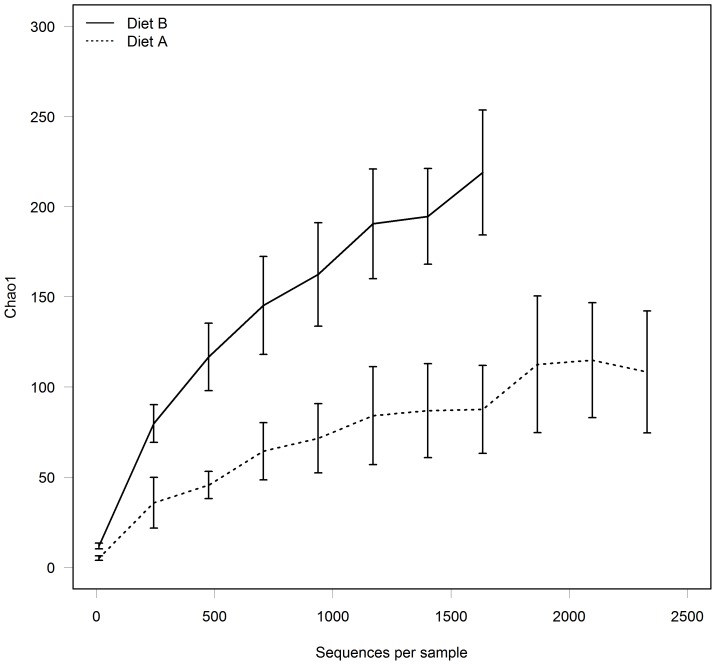
The effects of Diet A or Diet B on faecal microbial diversity in pregnant queens (*Felis catus*). The rarefaction curves based on the Chao1 diversity index (at 97% sequence identity cut-off) indicate that faecal bacterial communities of cats fed Diet A (---; n =  3 cats) were less diverse than those of cats fed Diet B (–; n = 4 cats). Data are reported as means ± SEM.

Diversity of the resident bacterial community in pregnant queens was increased by feeding Diet B, as shown by the Chao1 diversity estimator (Figure 2). Diet significantly affected the proportion of phyla observed in the faeces of pregnant queens, with decreased Fusobacteria and Bacteroidetes and increased Firmicutes observed in queens fed with the Diet A (Table 3). In pregnant queens fed Diet A, over 99% of the bacteria observed belonged to the phylum Firmicutes. In contrast, faeces from queens on Diet B contained Firmicutes (46.1% of total reads), Bacteroidetes (25.7% of total reads) and Fusobacteria (25.4% of total reads) as the predominant phyla. In total, 29 bacterial genera were observed in the faeces of the pregnant queens.

**Table 3:** The effect of diets on the bacterial phyla (% of total sequences) in faecal samples of pregnant female cats (*Felis catus*) fed Diet A (n = 3) or Diet B (n = 4).

Phylum	Diet A	Diet B			
	Mean	Mean	SEM	*P value*	*FDR*
Actinobacteria	0.21	0.12	0.07	0.47	1.00
Bacteroidetes	0.31	27.39	4.67	0.06	0.30
Firmicutes	99.26	46.12	4.34	0.01	0.04
Fusobacteria	0.14	24.98	2.18	0.01	0.05
Proteobacteria	0.03	1.02	0.27	0.15	0.74

*P value* indicates ANOVA significance of rank transformed data and False discovery Rate (*FDR)* indicates multiple testing adjusted *P* value.

### Influence of pre-weaning diet on faecal bacterial communities in kittens

Pre-weaning diet did not affect the proportion of phyla in the kitten faeces (Table 4), however, the proportions of some taxa were altered (Table 5). Kittens from mothers fed the Diet A showed increased abundance of *Solobacterium, Peptococcaceae* and *Clostridiales and Clostridium* (Table 5) and decreased abundance of Megamonas and Allisonella (Table 5).

**Table 4:** The effects of pre-weaning (gestation and lactation) or post-weaning diets (Diet A or B) on the bacterial phyla (proportion of total sequences) present in faecal samples of the domestic kitten (*Felis catus*; n = 5 per treatment).

Phylum	A –A Mean	A-B Mean	B-A Mean	B-B Mean	SEM	Pre-weaning P-value^1^	Pre-weaning FDR^1^	Post-weaning P-value^2^	Post-weaning FDR^2^	Pre * Post weaning P-value	Pre * Post weaning FDR
Actinobacteria	0.74	0.21	0.47	0.16	0.08	0.26	1.00	0.03	0.13	0.70	1.00
Bacteroidetes	18.83	20.08	18.60	26.92	3.90	0.53	1.00	0.15	0.74	0.31	1.00
Firmicutes	76.45	60.90	77.88	46.77	4.80	0.75	1.00	<0.001	0.02	0.28	1.00
Fusobacteria	1.66	13.24	1.62	24.14	1.54	0.83	1.00	<0.001	<0.001	0.02	0.12
Proteobacteria	1.97	4.85	1.21	1.38	0.81	0.91	1.00	0.21	1.00	0.79	1.00

Diet A-Diet A (A-A) n = 3 females, n = 2 males.

Diet A- Diet B (A-B) n = 3 females, n = 2 males.

Diet B- Diet A (B-A) n = 3 females, n = 2 males.

Diet B- Diet B (B-B) n = 4 females, n = 1 male.

1 Comparisons between Diets A-A and A-B vs B-A and B-B.

2 Comparison between Diets A-A and B-A and B-B and A-B.

Results represent an average of samples taken at week 8 and 17. *P value* indicates ANOVA significance of rank transformed data and False discovery Rate (*FDR)* indicates multiple testing adjusted *P* value.

**Table 5:** The effects of pre-weaning (gestation and lactation) or post-weaning (Diet A or Diet B) diets on the different bacterial genera (proportion of total sequences) present in at least 5 faecal samples from the domestic kitten (*Felis catus*; n = 5 per treatment) at a level of 0.5% or higher.

Phyla and Genera	A-A Mean	A-B Mean	B-A Mean	B-B Mean	SEM	Pre-wean P-value^1^	Pre-wean FDR^1^	Post-wean P-value^2^	Post-wean FDR^2^	Pre*Post P-value	Pre*Post FDR
**Actinobacteria**											
Collinsella	0.57	0.18	0.32	0.10	0.06	0.21	1.00	0.02	0.45	0.79	1.00
**Bacteroidetes**											
Prevotella	16.13	5.81	16.95	9.81	3.15	0.36	1.00	0.26	1.00	0.26	1.00
Unclassified Prevotellaceae	0.31	6.31	0.16	5.92	0.50	0.18	1.00	<0.01	0.00	0.68	1.00
Unclassified Bacteroidales	0.07	0.27	0.19	0.33	0.05	0.89	1.00	<0.01	0.00	0.63	1.00
Bacteroides	2.08	6.86	0.89	10.60	1.12	1.00	1.00	<0.01	0.00	0.14	1.00
**Firmicutes**											
Unclassified Firmicutes	0.50	0.28	0.47	0.20	0.06	0.14	1.00	0.01	0.26	0.78	1.00
Solobacterium	3.32	6.41	2.78	3.45	0.97	0.01	0.16	0.13	1.00	0.70	1.00
Unclassified Veillonellaceae	1.54	2.28	1.77	4.58	0.37	0.12	1.00	<0.01	0.10	0.11	1.00
Megasphaera	14.00	0.02	11.32	0.01	2.26	0.92	1.00	0.01	0.31	0.61	1.00
Megamonas	1.52	1.48	1.86	4.54	0.53	0.05	1.00	0.14	1.00	0.11	1.00
Allisonella	0.19	0.30	0.49	0.39	0.06	0.04	1.00	0.45	1.00	0.79	1.00
Acidaminococcus	0.43	0.00	1.01	0.00	0.13	0.53	1.00	<0.01	0.01	0.85	1.00
Unclassified Ruminococcaceae	0.38	2.67	0.76	2.06	0.31	0.58	1.00	<0.01	0.00	0.58	1.00
Unclassified Peptostreptococcaceae	10.35	31.85	12.22	21.46	3.54	0.49	1.00	0.02	0.43	0.87	1.00
Unclassified Peptococcaceae	0.40	0.70	0.32	0.31	0.08	0.03	0.83	0.22	1.00	0.30	1.00
Unclassified Clostridiales	1.93	4.98	1.81	2.93	0.34	0.05	1.00	<0.01	0.01	0.18	1.00
Unclassified Lachnospiraceae	1.19	2.22	1.39	2.12	0.29	0.86	1.00	0.02	0.58	0.27	1.00
Blautia	2.29	1.29	0.86	1.48	0.37	0.24	1.00	0.40	1.00	0.30	1.00
Clostridium	0.19	1.23	0.02	0.68	0.16	0.05	1.00	<0.01	<0.01	0.42	1.00
Streptococcus	28.62	0.04	9.08	0.09	3.45	0.21	1.00	0.01	0.14	0.08	1.00
Lactobacillus	4.68	1.07	17.72	0.27	1.92	0.16	1.00	<0.01	<0.01	0.67	1.00
Enterococcus	0.44	1.03	11.78	0.01	1.46	0.49	1.00	0.20	1.00	0.34	1.00
**Fusobacteria**											
Fusobacterium	1.48	10.56	0.44	20.13	1.17	0.48	1.00	<0.01	<0.01	0.01	0.18
Unclassified Fusobacteriaceae	0.17	2.59	1.18	3.55	0.56	0.70	1.00	<0.01	<0.01	0.29	1.00
**Proteobacteria**											
Sutterella	0.27	0.25	0.24	0.79	0.10	0.20	1.00	0.04	1.00	0.29	1.00

Diet A- Diet B (A-B) n = 3 females, n = 2 males.

Diet B- Diet A (B-A) n = 3 females, n = 2 males.

Diet B- Diet B (B-B) n = 4 females, n = 1 male.

1 Comparisons between Diets A-A and A-B vs B-A and B-B.

2 Comparison between Diets A-A and B-A and B-B and A-B.

Results represent an average of samples taken at week 8 and 17. *P value* indicates ANOVA significance of rank transformed data and False discovery Rate (*FDR)* indicates multiple testing adjusted *P* value. Diet A-Diet A (A-A) n = 3 females, n = 2 males.

### Influence of post-weaning diet format on faecal bacterial community composition of kittens

**Figure 3:**
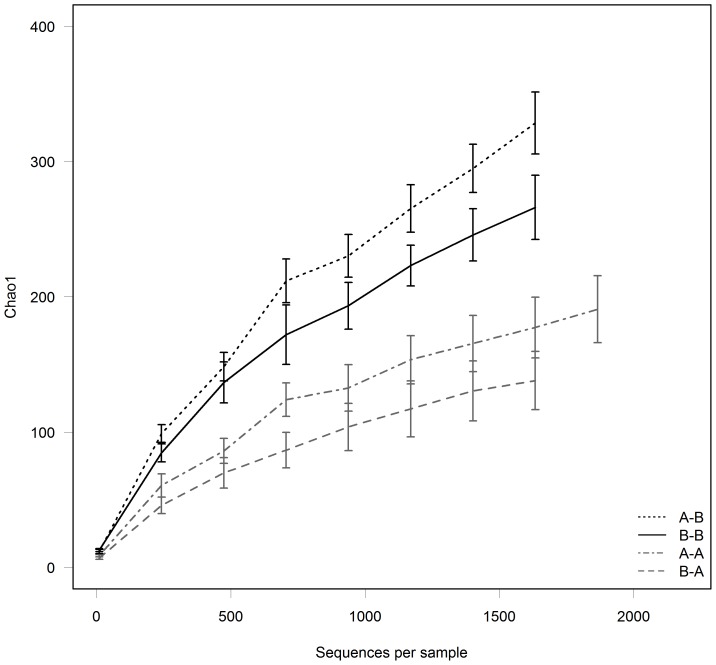
The effects of pre- and post-weaning feeding of Diet A or Diet B on faecal microbial diversity in kittens (*Felis catus*). The rarefaction curves based on the Chao1 diversity index (at 97% sequence identity cut-off) indicate that faecal bacterial communities of kittens fed Diet B (A-B and B-B; n = 10) were more diverse than those fed Diet A (A-A and B-A, n = 10). Pre-weaning diet did not have an effect on community diversity in the kitten. Data are reported as means ± SEM.

**Figure 4:**
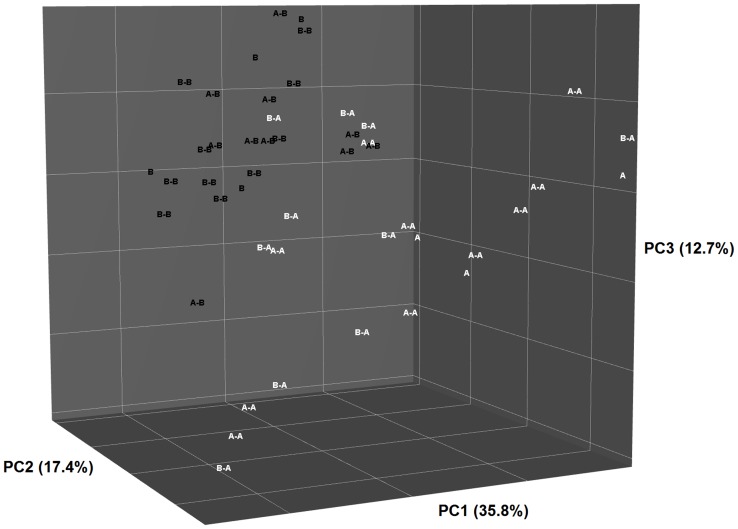
Principal Coordinate Analysis plot of weighted Unifrac phylogenetic distances showing the similarities between bacterial communities of queens fed Diet A or Diet B and their offspring fed Diet A (B-A or A-A) or Diet B (B-B or A-B) post-weaning. Percentage of variation captured by each component indicated on axes.

**Figure 5:**
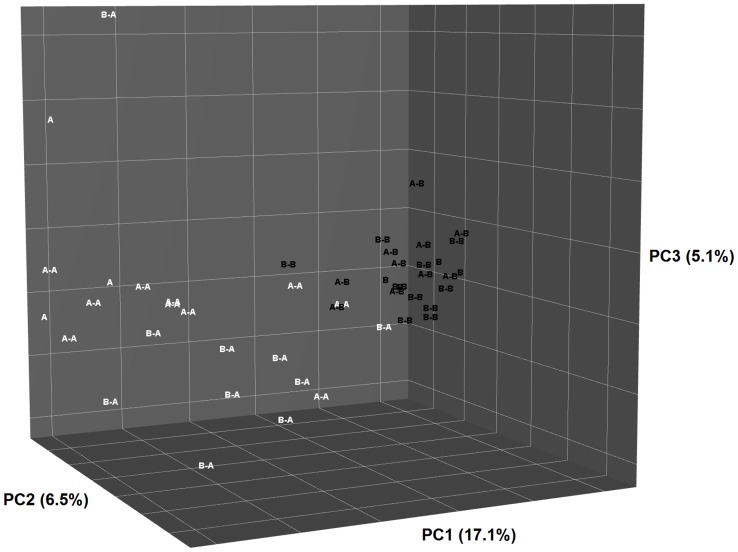
Principal Coordinate Analysis plot of unweighted Unifrac phylogenetic distances showing the similarities between bacterial communities of queens fed Diet A or Diet B and their offspring fed Diet A (B-A or A-A) or Diet B (B-B or A-B) post-weaning. Percentage of variation captured by each component indicated on axes.

There was no effect of sampling age on the microbial populations (8 or 17 wk, data not shown), therefore the results from these two periods were pooled. In kittens, the diversity of the bacterial population was affected by post-weaning diet (Figure 3). Principal Coordinate Analysis (PCoA) of weighted and unweighted Unifrac phylogenetic distances showed that the overall microbiota community structure was most similar between offspring fed with the same post-weaning diet, regardless of the mother’s diet (Figure 4 and 5). Multivariate analysis indicated that post weaning diet had a significant effect on community composition (P<0.001), while differences between pre-weaning diet tended towards significance (P = 0.07). No significant interaction between pre- and post-weaning diets were observed (P = 0.49).

Sequence types representing five different phyla were observed in the faeces of kittens. Firmicutes and Bacteriodetes were the most abundant phyla observed in the kittens irrespective of diet, followed by Fusobacteria and Proteobacteria, with sequences belonging to the phylum Actinobacteria were the least abundant. Dietary format affected the proportions of phyla with Firmicutes (77% of total reads), Bacteroidetes (19% of total reads) and Proteobacteria (1.6% of total reads) being predominant in kittens exposed to Diet A. In contrast Firmicutes (54% of total reads), Bacteroidetes (24% of total reads) and Fusobacteria (19% of total reads) being predominant in kittens exposed to Diet B. Post-weaning diet significantly affected (P < 0.05) the proportion of phyla, with decreased Fusobacteria and increased Firmicutes and Actinobacteria for kittens fed Diet A (Table 4).

In total, 19 bacterial families and 26 bacterial genera were identified in the faeces of kittens (Table 5). Post-weaning dietary format significantly affected 19 genera (Table 5). Predominant genera for the kittens fed Diet A were *Streptococcus* (18.9% of total reads), *Prevotella* (16.5% of total reads) and *Megaspheara* (12.7% of total reads). For kittens fed Diet B, the most dominant genera were unclassified genera belonging to the families *Peptostreptococcaceae* (26.7% of total reads) and *Fusobacteriaceae* (15.4% of total reads). Major shifts relating to post-weaning diet included decreased percentage of sequences for *Bacteroides* and *Fusobacterium* and increased percentage of sequences for *Megasphaera*, *Streptococcus* and *Lactobacillus* for kittens fed Diet A post-weaning (Table 5).

### Influence of pre- and post-weaning diet on tissue gene expression in kittens

**Figure 6:**
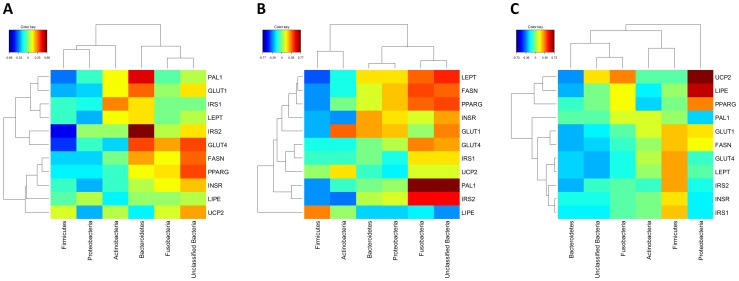
Correlation heatmaps of faecal microbiota and gene expression in (A) blood, (B) adipose fat tissue, and (C) reproductive tissue. Cell colour indicates strength and direction of correlation, with blue representing maximal negative correlation and red representing maximal positive correlation.

Maternal diet affected (P<0.05) the expression levels of LEPT, PAI-1 and INSR and tended (P<0.10) to affect the expression of GLUT1 (Table 6) in blood. In abdominal fat, expression levels of IRS-1 was affected by pre-weaning diet (P<0.05). There was no effect of pre-weaning diet in the expression levels of genes in reproductive tissues. There was no effect (P>0.05) of post-weaning diet on gene expression levels in the kittens fed either diet (Table 6). Despite only limited numbers of genes showing differential expression due to dietary treatment, strong correlations were observed between the various members of the microbiota and gene expression in blood, fat and reproductive tissue (Figure 6). Abundance of *Firmicutes* was negatively correlated with expression of most genes examined in blood and fat, whereas abundance of *Bacteroidetes* tended to be positively correlated with gene expression in blood and fat. In contrast, the correlation between *Bacteroidetes* and *Firmicutes* abundance and gene expression levels showed the opposite pattern in reproductive tissue, with the *Bacteroidetes* showing a negative correlation with gene expression, while the *Firmicutes* showed a positive correlation. The correlation profiles of the *Actinobacteria* abundance and gene expression levels were also most closely similar to that of the *Firmicutes* compared to other bacterial groups.

**Table 6:** The effects of pre-weaning (gestation and lactation) or post-weaning diets (Diet A or Diet B) on blood and tissue gene expression levels (relative fluorescence units) in the domestic kitten (*Felis catus*).

Tissue	Gene	D-D Mean	D-W Mean	W-D Mean	W-W Mean	SEM	Pre-weaning *P* value^2^	Pre-weaning *FDR* 2	Post-weaning *P* value^3^	Post-weaning *FDR* 3	Pre*Postweaning *P* value	Pre*Postweaning *FDR*
**Blood** 4												
	FASN	0.84	1.11	1.31	1.29	0.48	0.11	1.00	0.52	1.00	0.45	1.00
	GLUT1	4.26	7.63	11.51	11.40	4.00	0.08	0.88	0.58	1.00	0.56	1.00
	GLUT4	26.64	39.72	44.11	55.19	18.12	0.12	1.00	0.25	1.00	0.92	1.00
	INSR	5.31	8.05	8.97	10.21	3.39	0.03	0.33	0.13	1.00	0.56	1.00
	IRS-1	2.96	1.74	3.11	3.51	1.21	0.16	1.00	0.54	1.00	0.24	1.00
	IRS-2	0.28	0.16	0.33	0.29	0.12	0.26	1.00	0.32	1.00	0.67	1.00
	LEPT	13.12	21.24	29.48	33.96	10.96	0.03	0.33	0.32	1.00	0.77	1.00
	LIPE	2.56	2.86	2.88	2.16	1.15	0.85	1.00	0.83	1.00	0.61	1.00
	PAI-1	0.14	0.16	0.44	0.36	0.12	<0.001	0.01	0.60	1.00	0.41	1.00
	PPARG	3.19	4.97	5.32	4.79	1.90	0.28	1.00	0.48	1.00	0.20	1.00
	UCP2	7.44	10.45	6.45	7.34	3.11	0.27	1.00	0.29	1.00	0.56	1.00
**Abdominal fat^5^**												
	FASN	4.60	4.31	1.59	4.27	1.90	0.29	1.00	0.37	1.00	0.31	1.00
	GLUT1	0.55	0.62	0.86	0.64	0.32	0.61	1.00	0.81	1.00	0.67	1.00
	GLUT4	17.09	20.17	6.96	17.37	7.29	0.16	1.00	0.12	1.00	0.40	1.00
	INSR	2.35	2.69	2.17	2.24	1.12	0.58	1.00	0.72	1.00	0.80	1.00
	IRS-1	0.62	0.42	0.25	0.32	0.17	0.05	0.55	0.59	1.00	0.25	1.00
	IRS-2	0.28	0.35	0.20	0.36	0.14	0.75	1.00	0.19	1.00	0.62	1.00
	LEPT	19.76	27.86	14.07	23.47	10.31	0.54	1.00	0.24	1.00	0.93	1.00
	LIPE	2.55	3.29	39.36	2.86	6.08	0.39	1.00	0.37	1.00	0.38	1.00
	PAI-1	0.26	0.57	0.25	0.60	0.20	0.86	1.00	0.11	1.00	0.93	1.00
	PPARG	5.35	4.51	2.31	4.94	2.09	0.38	1.00	0.51	1.00	0.27	1.00
	UCP2	1.83	2.23	1.37	1.55	0.85	0.29	1.00	0.57	1.00	0.83	1.00
**Reproductive organs** 6												
	FASN	0.66	0.67	1.74	0.41	0.37	0.54	1.00	0.33	1.00	0.32	1.00
	GLUT1	0.15	0.16	0.28	0.11	0.07	0.60	1.00	0.32	1.00	0.30	1.00
	GLUT4	2.77	2.93	20.60	1.89	3.07	0.40	1.00	0.35	1.00	0.34	1.00
	INSR	5.90	3.37	2.33	4.02	1.95	0.45	1.00	0.83	1.00	0.28	1.00
	IRS-1	0.67	0.73	1.04	0.85	0.33	0.46	1.00	0.85	1.00	0.72	1.00
	IRS-2	0.30	0.80	0.75	0.49	0.25	0.83	1.00	0.73	1.00	0.27	1.00
	LEPT	0.14	0.11	0.79	0.14	0.12	0.36	1.00	0.36	1.00	0.40	1.00
	LIPE	2.28	2.62	2.32	1.30	0.93	0.62	1.00	0.79	1.00	0.61	1.00
	PAI-1	0.27	0.19	0.21	0.33	0.11	0.65	1.00	0.81	1.00	0.29	1.00
	PPARG	1.34	1.60	2.58	2.17	0.87	0.42	1.00	0.94	1.00	0.77	1.00
	UCP2	3.26	3.31	2.42	2.42	1.11	0.36	1.00	0.98	1.00	0.97	1.00

Diet A-Diet A (A-A) n = 3 females, n = 2 males.

Diet A- Diet B (A-B) n = 3 females, n = 2 males.

Diet B- Diet A (B-A) n = 3 females, n = 2 males.

Diet B- Diet B (B-B) n = 4 females, n = 1 male.

1 Fatty acid synthase (FASN), Glucose transporter 1 (Glut1) and 4 (Glut4), insulin receptor (INSR), insulin receptor substrate 1 (IRS1) and 2 (IRS2), Leptin (LEPT), Hormone sensitive lipase (LIPE), plasminogen activator inhibitor-1 (PAL1), Uncoupling protein 2 (UCP2), peroxisome proliferative activated receptor-γ (PPARG).

2 Comparisons between Diets A-A and A-B vs B-A and B-B.

3 Comparison between Diets A-A and B-A and B-B and A-B.

4 n = 5 per treatment.

5. n = 4 per treatment.

6 Ovarian or testicular tissue.

Results are presented as means ± SEM. *P* indicates ANOVA significance of rank transformed data and *FDR* indicates multiple testing adjusted *P* value. No effect of gender was observed.

## Discussion

We hypothesised that pre-weaning diet would significantly impact the composition of the faecal microbiota of the kitten. This study shows that despite high faecal microbial diversity in pregnant queens, the pre-weaning diet had no effect on faecal microbiota composition in offspring, but gene expression levels in blood and adipose tissues were altered by pre-weaning diet. This suggests that the maternal influences on gene expression are long lasting whereas its effects on microbial populations may be relatively short-term despite their relative stability over the lifetime of the individual [7], [10].

Previous studies in rodents suggest that pre-weaning diet has an effect on the microbiota of the offspring, especially in early life [21], [32]. The current study using next generation sequencing identified that pre-weaning diet had a small impact on the composition of the microbiota in the intestinal tract of the kittens, with post-weaning diets having a major role in influencing the composition of the faecal microbiota. This is evidenced by the large differences in the distribution of phyla between the pregnant queens and their offspring. In the current study, the pre-weaning period included both gestation and lactation and the transition of the offspring from milk to solid food. The pre-weaned offspring were therefore exposed to milk from their mother as well as their post-weaning diet. Bacterial species composition of the milk was determined using both culture-dependent and independent methodologies (Cookson, unpublished data) and suggested increased species diversity in milk from cats fed Diet A. Despite this we did not see many changes in intestinal microbial composition that were attributed to pre-weaning diet. In contrast, post-weaning diet had a major impact on the faecal microbiota of the analysed kittens.

Kittens fed Diet A post weaning had increased abundance of Firmicutes and Actinobacteria and decreased abundance of Fusobacteria compared to kittens fed Diet B post-weaning, consistent with results observed in adult cats fed similar diets [9] and the queens in this study. The majority of studies investigating microbes in healthy mammals have identified Firmicutes and Bacteroidetes as the predominant bacterial populations present in the intestinal tracts. Our study corroborates these results. However, the predominant phyla detected in this study, Firmicutes and Bacteroidetes, differed to those observed previously [32] who reported Firmicutes and Actinobacteria as the two most abundant phyla.

Actinobacteria showed a decreased abundance in kittens fed Diet B post-weaning. Included in the Actinobacteria are members of the genus *Bifidobacterium* and the family *Coriobacteriaceae*. Both of these taxa are associated with improved health in rodent models. For example, Bifidobacteria are thought to improve intestinal health [33], while *Coriobacteriaceae* are associated with decreased blood glucose and cholesterol metabolism in rodents [34], [35]. In the current study, *Collinsella* was significantly reduced in the kittens fed Diet B. Decreased *C. aerofaciens* has been associated with low carbohydrate diets in human subjects [36]. The overall proportion of Actinobacteria in the current study (averaging 0.6%) was much lower than that reported previously for kittens (28.5%) [32] and adult cats (16%) [9]) fed similar diets. Bifidobacteria were not detected in our study, contrary to a previous study investigating the faecal microbiome of kittens fed high-protein (52% DM) diets [32]. Results from our current study are in agreement with an earlier study from our laboratory using adult cats [9], but differ from previous studies [32], probably due to differences in analytical methodologies (primer design etc).

Bacteroidetes are associated with both protein and CHO digestion [37]. In the current study there were no differences in the abundance of members of the Bacteroidetes associated with diet; this agrees with results in adult cats fed similar diets [9]. Within the Bacteroidetes, the abundance of *Bacteroides* was higher in kittens fed Diet B diet post-weaning. In the current study, the abundance of *Bacteroides* was also higher in kittens fed the Diet B post-weaning. This contrasts with results in adult cats which showed a decrease of *Bacteroides* associated with a similar diet [9]. Bacteroidete proportions were higher in the current study (c. 20%) compared to that observed previously in kittens fed moderate or high protein:carbohydrate dry diets (0.2% [32]).

Kittens fed Diet B had lower abundance of Firmicutes compared to kittens fed Diet A. The abundance of Firmicutes in kittens fed Diet A post-weaning were similar to those reported in kittens of the same age fed a dry, moderate protein:CHO diet [32]. Within the phylum Firmicutes, large shifts in the abundances of *Lactobacillaceae* (*Lactobacillus*), *Veillonellaceae* (*Megasphaera*), *Peptostreptococcaceae* (*Unclassified Peptostreptococcaceae*), and *Streptococcaceae* (*Streptococcus*) were observed between diets. *Lactobacillus* populations were higher in kittens fed Diet A compared to kittens fed Diet B. Increased *Lactobacillus* on Diet A is in agreement with results in kittens fed a dry, moderate protein:CHO diets [32] and adult cats fed dry diets [9]. Similar to [32] who observed increased *Megasphaera* associated with a moderate protein:CHO diet, *Megasphaera* spp. were also more abundant in kittens fed Diet A in the current study. It is possible that the increased *Megasphaera* proportions were due to the increase in *Lactobacillus* spp. observed in the current study. In contrast, *Peptostreptococcaceae* proportions were higher in kittens on Diet B, which was in agreement with results in adult cats [9].

Fusobacteria are proteolytic bacteria [38] and are seen in low levels of in healthy cats (0.3-5% of sequences) [32], [39], [40]. They have been associated with high protein diets in kittens [32], adult cats [9] and dogs [41]. In the current study, the abundance of phylotypes belonging to the order *Fusobacterium* was higher in kittens fed Diet B versus Diet A post-weaning (15 *vs.* 0.9%, respectively).

Overall, Proteobacteria proportions were not different between kittens fed Diet A or Diet B, with only *Sutterella* spp. increased in kittens fed Diet B post-weaning. Previous research has shown increased Proteobacteria associated with a dry, high protein:carbohydrate diet in kittens [32] and wet diets in adult cats [9]. The protein levels in the current study were similar (35% vs. 45% in Diets A and B, respectively) to that fed to adult cats (32–42%) [9].

In recent years there has been increasing focus on the effects of diet on the intestinal microbiota and the role they might play in the health of the host, including the domestic cat. However, much of the research carried out in the domestic cat has been conducted using research-formulated diets, which are generally dry in nature. In the current study, the macronutrient contents of Diets A and B differed largely in fat (37.6% vs. 20.2% respectively) and carbohydrate (2.0% vs. 28.2% respectively) with protein being slightly higher in Diet B (45.3% vs. 35.3% respectively). Because of these differences we are unable to make any conclusions about the type(s) of nutrient(s) that might have caused the shifts in bacterial abundances observed, however, macronutrient digestibility (i.e., protein, fat) was decreased in kittens on Diet A (data not shown). However, in rodent models Proteobacteria and Firmicutes increased in association with high fat diets [42]. The diets in the current study also differed in their protein source. In rodent models, beef and milk (standard chow) proteins affected the phylogenetic composition and diversity of intestinal bacterial communities [43], [44], with increased diversity associated with the beef-based diets. Increased bacterial diversity has been observed in adult cats fed a meat-based diet (Diet B) [9]. It is therefore plausible that protein source may also affect bacterial community composition in the cat. The different effects of high protein diets on *Fusobacteria* and *Lactobacillus* spp. in different hosts may therefore reflect evolutionary differences in diet preference and is of interest for future research.

Due in part to the rise of obesity in the domestic cats, there is increasing literature on the effects of obesity [45] and insulin sensitivity [28], [46], [47] on gene expression in the domestic cat. Obesity in the domestic cat has been linked to decreased IRS-1 and IRS-2 expression levels in liver and skeletal muscle [28]. IRS-1 is involved in insulin resistance and is involved in the regulation of glucose uptake and conversion into fat cells [47]. While obesity is also linked with decreased GLUT4 protein expression levels [45] lipid-infused cats show increased GLUT4 mRNA expression levels [46]. While bodyweight gain rather than diet is thought to increase the occurrence of pre-diabetic state in the domestic cat [48], the effects of maternal diet on gene expression in the domestic cat are of interest due to the long-term effects that these changes may have in relation to the health outcomes of the offspring [6]. In the current study, offspring fed Diet B pre-weaning increased the expression levels of many genes including those that are involved in the glucose (GLUT1, INSR) and lipid (LEPT, PAI-1) metabolic pathways and decreased IRS-1 expression levels in abdominal fat. Previous studies in kittens have shown that high protein, pre-weaning diets increases the expression levels of key genes in the insulin (e.g., IR), glucose (e.g., uncoupling protein 2) and lipid (e.g., LEPT) metabolic pathways [6].

There is increasing interest in the interaction between the host and its intestinal microbiota profiles and how they interact. In this study, Firmicutes and Bacteroidetes had opposite correlations with expression of genes in the glucose, insulin and lipid metabolic pathways. For example, in blood, Firmicutes are negatively correlated to glucose and insulin genes, whereas Bacteriodetes are positively correlated. However, in reproductive tissues Firmicutes are positively correlated to glucose and insulin genes and Bacteriodetes are negatively correlated. It is established in rodent and human that the ratio of Firmicutes:Bacteriodetes increases in obese humans and rodents [12], [13], [49], [50]. This ratio has been associated in perturbations in energy homeostasis and the development of insulin resistance (see [51], [52] and may explain the observations of the current study. In abdominal fat tissue, fusobacteria abundance was strongly correlated with LEPT, FASN, PPARG, PAI-1 and IRS-2 gene expression levels while it is was negatively correlated with LIPE. This suggests that Fusobacteria could also be a potential biomarker of perturbations in energy homeostasis and warrants further investigation. Furthermore, the *Firmicutes* and *Actinobacteria* had similar correlations with gene expression, suggesting overlapping or synergistic functions of these two taxa in the microbial community.

In conclusion this study has identified that while pre-weaning diet plays a role in the alteration of gene expression levels it has little impact on the faecal bacterial profiles of the offspring. In contrast, while post-weaning diet format has no effect on gene expression levels in offspring, it plays a critical role in faecal bacterial composition. Why this may be the case is of interest for future research. Relationships exist between intestinal microbiota profiles and genes involved in the glucose and insulin pathways and these may play a role in long-term health.

## References

[1] Low FM, Gluckman PD, Hanson MA (2011) Developmental plasticity and epigenetic mechanisms underpinning metabolic and cardiovascular diseases. Epigenomics 3: 279–294. 22122338 10.2217/epi.11.17

[2] Gluckman PD, Hanson MA, Low FM (2011) The role of developmental plasticity and epigenetics in human health. Birth Defects Res C Embryo Today 93: 12–18. 21425438 10.1002/bdrc.20198

[3] Roseboom TJ, Watson ED (2012) The next generation of disease risk: Are the effects of prenatal nutrition transmitted across generations? Evidence from animal and human studies. Placenta. 10.1016/j.placenta.2012.07.018 22902003

[4] Liang C, Oest ME, Prater MR (2009) Intrauterine exposure to high saturated fat diet elevates risk of adult-onset chronic diseases in C57BL/6 mice. Birth Defects Res B Dev Reprod Toxicol 86: 377–384. 19750488 10.1002/bdrb.20206

[5] Vucetic Z, Kimmel J, Totoki K, Hollenbeck E, Reyes TM (2010) Maternal high-fat diet alters methylation and gene expression of dopamine and opioid-related genes. Endocrinology 151: 4756–4764. 20685869 10.1210/en.2010-0505 PMC2946145

[6] Vester BM, Liu KJ, Keel TL, Graves TK, Swanson KS (2009) In utero and postnatal exposure to a high-protein or high-carbohydrate diet leads to differences in adipose tissue mRNA expression and blood metabolites in kittens. Br J Nutr 102: 1136–1144. 19445818 10.1017/S0007114509371652

[7] Lozupone CA, Stombaugh JI, Gordon JI, Jansson JK, Knight R (2012) Diversity, stability and resilience of the human gut microbiota. Nature 489: 220–230. 22972295 10.1038/nature11550 PMC3577372

[8] Bermingham EN, Kittelmann S, Young W, Henderson G, Roy NC, et al (2011) Short-term feeding of wet and dry diets alters the faecal bacterial populations in the domestic cat (*Felis catus*). British Journal of Nutrition 106: s49–s52. 22005435 10.1017/S0007114511000572

[9] Bermingham EN, Young W, Kittelmann S, Kerr KR, Swanson KS, et al (2013) Dietary format alters fecal bacterial populations in the domestic cat (Felis catus). MicrobiologyOpen DOI: 10.1002/mbo3.60 10.1002/mbo3.60 PMC3584222 23297252

[10] Zoetendal EG, Akkermans AD, De Vos WM (1998) Temperature gradient gel electrophoresis analysis of 16S rRNA from human fecal samples reveals stable and host-specific communities of active bacteria. Applied and Environmental Microbiology 64: 3854–3859. 9758810 10.1128/aem.64.10.3854-3859.1998 PMC106569

[11] De La Serre CB, Ellis CL, Lee J, Hartman AL, Rutledge JC, et al (2010) Propensity to high fat diet-induced obesity in rats is associated with changes in the gut microbiota and gut inflammation. Am J Physiol Gastrointest Liver Physiol 299: G440–G448. 20508158 10.1152/ajpgi.00098.2010 PMC2928532

[12] Turnbaugh PJ, Hamady M, Yatsunenko T, Cantarel BL, Duncan A, et al (2009) A core gut microbiome in obese and lean twins. Nature 457: 480–484. 19043404 10.1038/nature07540 PMC2677729

[13] Turnbaugh PJ, Backhed F, Fulton L, Gordon JI (2008) Diet-induced obesity is linked to marked but reversible alterations in the mouse distal gut microbiome. Cell Host Microbe 3: 213–223. 18407065 10.1016/j.chom.2008.02.015 PMC3687783

[14] Young W, Roy NC, Lee J, Lawley B, Otter D, et al (2012) Changes in bowel microbiota induced by feeding weanlings resistant starch stimulate transcriptomic and physiological responses. Appl Environ Microbiol 78: 6656–6664. 22798356 10.1128/AEM.01536-12 PMC3426708

[15] Round JL, O'Connell RM, Mazmanian SK (2010) Coordination of tolerogenic immune responses by the commensal microbiota. J Autoimmun 34: J220–225. 19963349 10.1016/j.jaut.2009.11.007 PMC3155383

[16] Sanz Y (2011) Gut microbiota and probiotics in maternal and infant health. Am J Clin Nutr 94: 2000S–2005S. 21543533 10.3945/ajcn.110.001172

[17] Neu J, Rushing J (2011) Cesarean versus vaginal delivery: long-term infant outcomes and the hygiene hypothesis. Clin Perinatol 38: 321–331. 21645799 10.1016/j.clp.2011.03.008 PMC3110651

[18] Thum C, Cookson AL, Otter DE, McNabb WC, Hodgkinson AJ, et al (2012) Can nutritional modulation of maternal intestinal microbiota influence the development of the infant gastrointestinal tract? Journal of Nutrition 142: 1921–1928. 22990463 10.3945/jn.112.166231

[19] Fardini Y, Chung P, Dumm R, Joshi N, Han YW (2010) Transmission of diverse oral bacteria to murine placenta: evidence for the oral microbiome as a potential source of intrauterine infection. Infect Immun 78: 1789–1796. 20123706 10.1128/IAI.01395-09 PMC2849412

[20] Fujiwara R, Takemura N, Watanabe J, Sonoyama K (2010) Maternal consumption of fructo-oligosaccharide diminishes the severity of skin inflammation in offspring of NC/Nga mice. Br J Nutr 103: 530–538. 19857365 10.1017/S000711450999198X

[21] Schaible TD, Harris RA, Dowd SE, Smith CW, Kellermayer R (2011) Maternal methyl-donor supplementation induces prolonged murine offspring colitis susceptibility in association with mucosal epigenetic and microbiomic changes. Hum Mol Genet 20: 1687–1696. 21296867 10.1093/hmg/ddr044 PMC3115577

[22] Karlsson CL, Molin G, Fak F, Johansson Hagslatt ML, Jakesevic M, et al (2011) Effects on weight gain and gut microbiota in rats given bacterial supplements and a high-energy-dense diet from fetal life through to 6 months of age. Br J Nutr 106: 887–895. 21450114 10.1017/S0007114511001036

[23] Hendriks WH, Wamberg S, Tarttelin MF (1999) A metabolism cage for quantitative urine collection and accurate measurement of water balance in adult cats (*Felis catus*). Journal of Animal Physiology and Animal Nutrition 82: 94–105.

[24] Kittelmann S, Janssen PH (2010) Characterisation of rumen ciliate community composition in domestic sheep, deer, and cattle feeding on varying diets, by means of PCR-DGGE and clone libraries. FEMS Microbiology Ecology 75: 468–481. 10.1111/j.1574-6941.2010.01022.x 21204869

[25] Fierer N, Hamady M, Lauber CL, Knight R (2008) The influence of sex, handedness, and washing on the diversity of hand surface bacteria. Proc Natl Acad Sci U S A 105: 17994–17999. 19004758 10.1073/pnas.0807920105 PMC2584711

[26] Weisburg WG, Barns SM, Pelletier DA, Lane DJ (1991) 16S ribosomal DNA amplification for phylogenetic study. J Bacteriol 173: 697–703. 1987160 10.1128/jb.173.2.697-703.1991 PMC207061

[27] Lane DJ, Pace B, Olsen GJ, Stahl DA, Sogin ML, et al (1985) Rapid determination of 16S ribosomal RNA sequences for phylogenetic analyses. Proc Natl Acad Sci U S A 82: 6955–6959. 2413450 10.1073/pnas.82.20.6955 PMC391288

[28] Mori A, Lee P, Takemitsu H, Iwasaki E, Kimura N, et al (2009) Decreased gene expression of insulin signaling genes in insulin sensitive tissues of obese cats. Veterinary Research Communications 33: 315–329. 18946721 10.1007/s11259-008-9179-y

[29] Zini E, Linscheid P, Franchini M, Kaufmann K, Monnais E, et al (2009) Partial sequencing and expression of genes involved in glucose metabolism in adipose tissues and skeletal muscle of healthy cats. The Veterinary Journal 180: 66–70. 18078768 10.1016/j.tvjl.2007.10.022

[30] Caporaso JG, Kuczynski J, Stombaugh J, Bittinger K, Bushman FD, et al (2010) QIIME allows analysis of high-throughput community sequencing data. Nat Methods 7: 335–336. 20383131 10.1038/nmeth.f.303 PMC3156573

[31] Wang Q, Garrity GM, Tiedje JM, Cole JR (2007) Naive Bayesian classifier for rapid assignment of rRNA sequences into the new bacterial taxonomy. Appl Environ Microbiol 73: 5261–5267. 17586664 10.1128/AEM.00062-07 PMC1950982

[32] Hooda S, Vester-Boler BM, Kerr KR, Dowd SE, Swanson KS (2012) The gut microbiome of kittens is affected by dietary protein: carbohydrate ratio and correlated with blood metabolite and hormone concentrations. British Journal of Nutrition. 10.1017/S0007114512003479 22935193

[33] Turroni F, van Sinderen D, Ventura M (2009) Bifidobacteria: from ecology to genomics. Front Biosci 14: 4673–4684. 10.2741/3559 19273381

[34] Claus SP, Ellero SL, Berger B, Krause L, Bruttin A, et al (2011) Colonization-induced host-gut microbial metabolic interaction. mBio 2: e00271–00210. 21363910 10.1128/mBio.00271-10 PMC3045766

[35] Martinez I, Wallace G, Zhang C, Legge R, Benson AK, et al (2009) Diet-induced metabolic improvements in a hamster model of hypercholesterolemia are strongly linked to alterations of the gut microbiota. Appl Environ Microbiol 75: 4175–4184. 19411417 10.1128/AEM.00380-09 PMC2698331

[36] Walker AW, Ince J, Duncan SH, Webster LM, Holtrop G, et al (2011) Dominant and diet-responsive groups of bacteria within the human colonic microbiota. ISME J 5: 220–230. 20686513 10.1038/ismej.2010.118 PMC3105703

[37] Thomas Fo, Hehemann J-H, Rebuffet E, Czjzek M, Michel G (2011) Environmental and gut Bacteroidetes: the food connection. Frontiers in Microbiology 2. 10.3389/fmicb.2011.00093 PMC3129010 21747801

[38] Loesche WJ, Gibbons RJ (1968) Amino acid fermentation by Fusobacterium nucleatum. Arch Oral Biol 13: 191–202. 5238887 10.1016/0003-9969(68)90051-4

[39] Ritchie LE, Steiner JM, Suchodolski JS (2008) Assessment of microbial diversity along the feline intestinal tract using 16S rRNA gene analysis. FEMS Microbiology Ecology 66: 590–598. 19049654 10.1111/j.1574-6941.2008.00609.x

[40] Handl S, Dowd SE, Garcia-Mazcorro JF, Steiner JM, Suchodolski JS (2011) Massive parallel 16S rRNA gene pyrosequencing reveals highly diverse fecal bacterial and fungal communities in healthy dogs and cats. FEMS Microbiol Ecol 76: 301–310. 21261668 10.1111/j.1574-6941.2011.01058.x

[41] Beloshapka AN, Dowd SE, Duclos L, Swanson KS (2011) Comparison of fecal microbial communities of healthy adult dogs fed raw meat-based or extruded diets using 454 pyrosequencing. Journal of Animal Science 89: 284.

[42] Hildebrandt MA, Hoffmann C, Sherrill-Mix SA, Keilbaugh SA, Hamady M, et al (2009) High-fat diet determines the composition of the murine gut microbiome independently of obesity. Gastroenterology 137: 1716–1724. 19706296 10.1053/j.gastro.2009.08.042 PMC2770164

[43] Bedani R, Pauly-Silveira ND, Roselino MN, de Valdez GF, Rossi EA (2010) Effect of fermented soy product on the fecal microbiota of rats fed on a beef-based animal diet. J Sci Food Agric 90: 233–238. 20355036 10.1002/jsfa.3800

[44] Li W, Dowd SE, Scurlock B, Acosta-Martinez V, Lyte M (2009) Memory and learning behavior in mice is temporally associated with diet-induced alterations in gut bacteria. Physiol Behav 96: 557–567. 19135464 10.1016/j.physbeh.2008.12.004

[45] Brennan CL, Hoenig M, Ferguson DC (2004) GLUT4 but not GLUT1 expression decreases early in the development of feline obesity. Domestic Animal Endocrinology 26: 291–301. 15063922 10.1016/j.domaniend.2003.11.003

[46] Zini E, Osto M, Konrad D, Franchini M, Sieber-Ruckstuhl NS, et al (2010) 10-day hyperlipidemic clamp in cats: effects on insulin sensitivity, inflammation, and glucose metabolism-related genes. Horm Metab Res 42: 340–347. 20162504 10.1055/s-0030-1248251

[47] Mori A, Lee P, Takemitsu H, Sako T, Arai T (2009) Comparison of insulin signaling gene expression in insulin sensitive tissues between cats and dogs. Vet Res Commun 33: 211–226. 19043794 10.1007/s11259-008-9168-1

[48] Backus RC, Cave NJ, Ganjam VK, Turner JBM, Biourge VC (2010) Age and body weight effects on glucose and insulin tolerance in colony cats maintained since weaning on high dietary carbohydrate. Journal of Animal Physiology and Animal Nutrition 94: e318–e328. 20626501 10.1111/j.1439-0396.2010.01014.x

[49] Ley RE, Turnbaugh PJ, Klein S, Gordon JI (2006) Microbial ecology: human gut microbes associated with obesity. Nature 444: 1022–1023. 17183309 10.1038/4441022a

[50] Turnbaugh PJ, Ley RE, Mahowald MA, Magrini V, Mardis ER, et al (2006) An obesity-associated gut microbiome with increased capacity for energy harvest. Nature 444: 1027–1031. 17183312 10.1038/nature05414

[51] Arora T, Sharma R (2011) Fermentation potential of the gut microbiome: implications for energy homeostasis and weight management. Nutr Rev 69: 99–106. 21294743 10.1111/j.1753-4887.2010.00365.x

[52] De Bandt JP, Waligora-Dupriet AJ, Butel MJ (2011) Intestinal microbiota in inflammation and insulin resistance: relevance to humans. Curr Opin Clin Nutr Metab Care 14: 334–340. 21587065 10.1097/MCO.0b013e328347924a

